# Clinical impact and prognostic implications of concurrent amyloid deposition in patients with POEMS syndrome: A single-center retrospective study

**DOI:** 10.1007/s00277-026-06995-1

**Published:** 2026-04-09

**Authors:** Yuhan Bao, Di Wang, Peiling Zhang, Ning An, Qiuxia Yu, Xinran Wang, Jianlin Hu, Chunrui Li

**Affiliations:** 1 Department of Hematology, Tongji Hospital of Tongji Medical College, Huazhong University of Science and Technology, 1095 Jie-Fang Avenue, Wuhan, Hubei 430030 P. R. China; 2 Key Laboratory of Vascular Aging, Tongji Hospital, Tongji Medical College, Ministry of Education, Huazhong University of Science and Technology, Wuhan, Hubei China

**Keywords:** POEMS syndrome, Amyloid deposition, Cardiac involvement, Biomarkers, Treatment response, Overall survival

## Abstract

POEMS syndrome is a rare paraneoplastic disorder driven by an underlying monoclonal plasma cell clone. A subset of patients develops concomitant amyloid deposition; however, the associated clinical profile and prognostic implications remain poorly defined. We retrospectively analyzed 62 patients with POEMS syndrome treated at Tongji Hospital between January 2018 and May 2025, comparing those with POEMS alone (*n* = 55) and those with biopsy-proven concurrent amyloid deposition (*n* = 7). Compared with POEMS-only patients, those with concurrent amyloid deposition were older (median 62 vs. 53 years; *p* = 0.022) and had higher IgA (6.21 vs. 2.17 g/L; *p* = 0.034) and λ free light chains (127.0 vs. 35.4 mg/L; *p* < 0.001). They also showed a more pronounced cardiac phenotype, including higher NT-proBNP (1250 vs. 359 pg/mL; *p* = 0.009) and hs-cTnI (47.4 vs. 2.1 pg/mL; *p* = 0.002), greater left ventricular wall thickness, and lower tissue Doppler velocities. Responses appeared numerically lower in patients with concurrent amyloid deposition, and overall survival was inferior compared with POEMS-only. In POEMS syndrome, biopsy-proven concurrent amyloid deposition marks a higher-risk phenotype characterized by disproportionate cardiac involvement and worse survival. These findings highlight the importance of clinical vigilance, careful evaluation for coexisting amyloid deposition, and multidisciplinary management.

**Supplementary Information:**

The online version contains supplementary material available at 10.1007/s00277-026-06995-1.

## Introduction

 POEMS syndrome (polyneuropathy, organomegaly, endocrinopathy, monoclonal plasma cell disorder, and skin changes) is a rare paraneoplastic syndrome secondary to an underlying plasma cell clone [1]. It is clinically heterogeneous, often marked by peripheral neuropathy, extravascular volume overload, and multisystem involvement [2]. Vascular endothelial growth factor (VEGF) is thought to play a key pathogenic role by promoting endothelial activation and capillary hyperpermeability, which contributes to edema, effusions, and organ dysfunction [3].

Systemic immunoglobulin light-chain (AL) amyloidosis is another clonal plasma cell disorder in which misfolded light chains aggregate into amyloid fibrils and deposit in tissues [4]. Cardiac and renal involvement are common and drive morbidity and mortality, with restrictive cardiomyopathy and nephrotic-range proteinuria representing major clinical presentations [5].

Although POEMS syndrome and amyloid deposition may rarely coexist, recognizing concomitant amyloid deposition in patients with POEMS syndrome can be challenging [6]. In such cases, overlapping manifestations (e.g., fluid retention, increased wall thickness, and biomarker elevation) may obscure recognition of amyloid deposition and delay appropriate risk stratification and management [7]. This distinction matters clinically because the major determinants of organ dysfunction, prognosis, and treatment approach may differ between POEMS syndrome and amyloidosis, particularly when cardiac involvement is present [8, 9]. Moreover, myocardial interstitial edema in POEMS syndrome may mimic hypertrophy, further complicating the recognition of true infiltrative cardiomyopathy [10]. Existing evidence is largely limited to isolated case reports and small series, leaving the prevalence, clinical phenotype, and prognostic implications of concurrent amyloid deposition in POEMS syndrome uncertain. To date, systematic cohort data comparing POEMS-only patients with those with biopsy-proven concurrent amyloid deposition remain scarce, especially those incorporating detailed cardiac phenotyping and longitudinal outcomes.

Here, we retrospectively analyzed a single-center cohort of patients with POEMS syndrome to estimate the frequency of biopsy-proven concurrent amyloid deposition, delineate its clinical and cardiac phenotype, and evaluate treatment responses and survival outcomes.

## Methods

### Study design and patients

This retrospective cohort study included consecutive patients with POEMS syndrome treated at Tongji Hospital (Tongji Medical College, Huazhong University of Science and Technology) between January 2018 and May 2025. POEMS syndrome and amyloidosis were diagnosed according to established Mayo Clinic criteria [11], with documentation of mandatory, major, and minor diagnostic components extracted from the medical record. Polyneuropathy was defined by compatible clinical features and/or electrophysiologic evidence when available. Amyloid deposition was confirmed histologically by Congo red positivity with apple-green birefringence under polarized light. Because formal amyloid fibril typing was not routinely available, amyloid subtype was not definitively assigned in this cohort. In patients with biopsy-proven amyloid deposition and coexisting monoclonal plasma cell disease, the clinicopathologic findings were interpreted in the context of an underlying plasma cell disorder [12]. ATTR amyloidosis was considered unlikely based on bone tracer cardiac scintigraphy findings, when available [13]. The study was approved by the Research Ethics Committee of Tongji Hospital (TJ-IRB202504070); informed consent was obtained using an opt-out approach in accordance with the Declaration of Helsinki.

### Clinical and laboratory assessment

All patients underwent standardized clinical evaluation and laboratory testing at diagnosis, including complete blood counts, comprehensive chemistry panels, serum immunoglobulins, and assessment of monoclonal proteins and serum free light chains. Cardiac assessment included electrocardiography and transthoracic echocardiography, with documentation of chamber dimensions, left ventricular wall thickness (including IVS and LVPW), left ventricular systolic function, diastolic indices, and tissue Doppler measurements. Cardiac biomarkers (NT-proBNP and hs-cTnI) were collected at baseline when available and interpreted in the clinical context of volume status. For clinical event ascertainment, heart failure was defined by documented clinician diagnosis and/or hospitalization for decompensated heart failure, and arrhythmias were defined as ECG- or monitoring-confirmed atrial or ventricular arrhythmias and clinically significant conduction disease documented in the medical record. Imaging, laboratory, treatment, and outcome data were extracted from electronic medical records using a prespecified case-report form.

### Response assessment

Hematologic response was categorized as complete (CRH), partial (PRH), or no response (NRH) based on changes in monoclonal protein and/or serum free light chains [14]. CRH required disappearance of detectable monoclonal protein by immunofixation (when available) with normalization of the free light-chain ratio in patients with measurable abnormalities. PRH required a substantial reduction in the involved light chain and/or M-protein relative to baseline. NRH was assigned when CRH/PRH criteria were not met. Clinical response of POEMS manifestations was graded as complete (CRC), partial (PRC), or no response (NRC) according to improvement in key syndrome-defining features documented during follow-up (including extravascular volume overload and other organ manifestations) using published criteria [14]. For patients with concurrent amyloid deposition, organ response was assessed according to consensus criteria as all-organ response (AOR), mixed organ response (MOR), or no organ response (NOR), with cardiac and renal responses evaluated when baseline involvement and follow-up measurements were available [15, 16].

### Follow-up and outcomes

Patients were followed from the time of POEMS diagnosis until death or the last clinical contact. Overall survival (OS) was defined as the time from diagnosis to death from any cause; patients who were alive at last follow-up were censored on that date.

### Statistical analysis

Categorical variables were compared using Fisher’s exact test, and continuous variables were compared using the Mann–Whitney U test. Overall survival (OS) was estimated using the Kaplan–Meier method and compared using the log-rank test. Univariate Cox proportional hazards models were used to explore associations between baseline variables and OS. For response analyses, the analysis population and denominator (standardized chemotherapy vs. supportive treatment only) were explicitly specified for each endpoint. Two-sided p values < 0.05 were considered statistically significant. Analyses were performed using R (version 4.5.2), and figures were generated using GraphPad Prism (version 8.0.5).

## Results

### Patient cohort

**Fig. 1:**
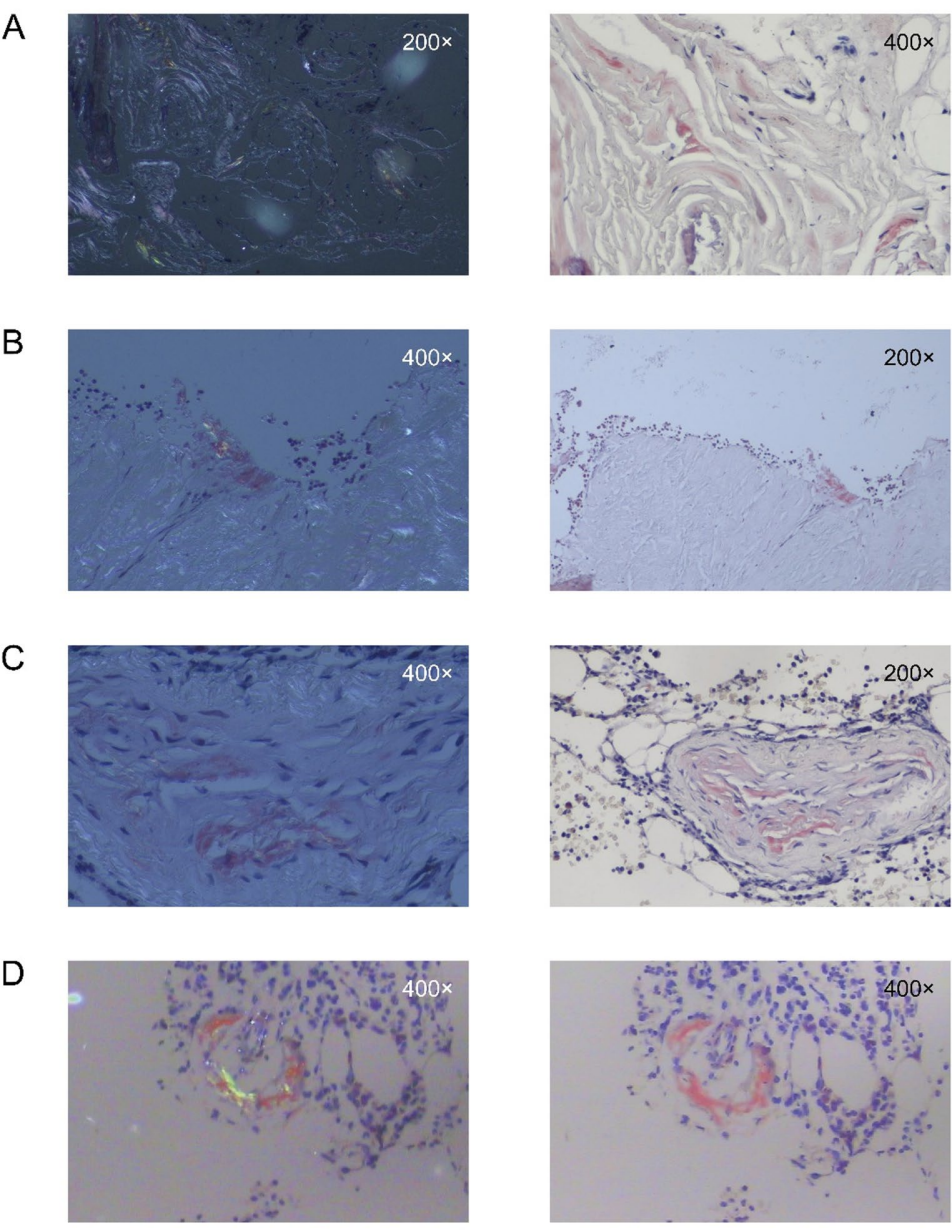
Representative Congo red staining of bone marrow specimens from patients with POEMS syndrome and concurrent amyloid deposition. Bone marrow specimens from four patients (**A–D**) was stained with Congo red. Images in the left column were obtained under polarized light, demonstrating apple-green birefringence, whereas those in the right column were obtained under bright-field microscopy. Magnifications are indicated in each panel

Between January 2018 and May 2025, consecutive patients with POEMS syndrome were screened for inclusion. A total of 62 patients met the eligibility criteria and were included in the final analysis (Supplementary Fig. 1). Seven patients (11.3%) had biopsy-proven concurrent amyloid deposition, while the remaining 55 patients constituted the POEMS-only group. Amyloid deposition was confirmed by Congo red staining with apple-green birefringence under polarized light, including bone marrow (*n* = 4) and kidney (*n* = 3) specimens; representative images are shown in Fig. 1. Baseline demographics and clinical characteristics are summarized in Table 1. The cohort showed a male predominance, consistent with prior reports from Asian POEMS populations [17].

**Table 1:** Baseline characteristics according to concurrent amyloid deposition status

Characteristic	Concurrent amyloid deposition (*n* = 7)	POEMS-only (*n* = 55)	*p* value
**Background**			
Age, years	62 (49–73)	53 (29–71)	**0.022**
Male sex, n (%)	6 (85.7)	39 (70.9)	0.408
**POEMS features**,** n (%)**			
Polyneuropathy	7 (100.0)	55 (100.0)	-
Organomegaly	4 (57.1)	36 (65.5)	0.665
Lymphadenopathy	3 (42.9)	34 (61.8)	0.335
Endocrinopathy	2 (28.6)	31 (56.4)	0.165
Any effusion	6 (85.7)	43 (78.2)	0.645
Pleural effusion	3 (42.9)	30 (54.5)	0.559
Ascites	1 (14.3)	15 (27.3)	0.460
Pelvic effusion	0 (0.0)	9 (16.4)	0.247
Pericardial effusion	6 (85.7)	30 (54.5)	0.115
Sclerotic bone lesions	2 (28.6)	26 (47.3)	0.349
VEGF, pg/mL	342.5 (129.9–783.5)	473.9 (11.0–10720.7)	0.565
Bone marrow plasma cells, %	1.0 (0.5–7)	1.0 (0.5–10)	0.925
M-protein, g/L	3.3 (0–10.9)	4.7 (0–19.1)	0.266
IgA isotype, n (%)	6 (85.7)	24 (48.0)	0.061
IgA, g/L	6.21 (0.07–9.89)	2.17 (0.38–19.10)	**0.034**
IgG, g/L	8.9 (5.9–23.9)	10.3 (3.0–30.9)	0.335
Lambda light chain, n (%)	7 (100.0)	43 (81.1)	0.208
Lambda free light chain, mg/L	127.0 (57.8–4861.0)	35.4 (9.5–1376.8)	**< 0.001**
Kappa free light chain, mg/L	18.5 (13.1–39.1)	33.8 (3.9–685.5)	0.105
**Laboratory findings**			
Hemoglobin, g/L	108 (93–160)	128 (76–170)	0.382
Platelets, ×10^9^/L	195 (99–422)	276.5 (21–592)	0.088
ALT, U/L	14 (4–26)	11 (4–43)	0.556
AST, U/L	20 (6–78)	14 (5–30)	0.133
Albumin, g/L	35.1 (32–39.3)	35.5 (18.6–46.2)	0.634
CRP, mg/L	1.1 (0.4–2.7)	2.6 (0.2–266.6)	0.340
Creatinine, µmol/L	104 (41–133)	77 (31–1037)	0.067
eGFR, mL/min/1.73 m²	61.1 (49.7–124.9)	98.0 (4.5–149.3)	0.054
NT-proBNP, pg/mL	1250 (159–20510)	359 (5–2512)	**0.009**
hs-cTnI, pg/mL	47.4 (1.9–3307.6)	2.1 (1.9–21.7)	**0.002**

Values are presented as median (range) or n (%), as appropriate. All measurements were obtained at baseline prior to systemic therapy. ‘-’ indicates that the p-value cannot be calculated

Abbreviations: ALT, alanine aminotransferase; AST, aspartate aminotransferase; CRP, C-reactive protein; eGFR, estimated glomerular filtration rate; hs-cTnI, high-sensitivity cardiac troponin I; NT-proBNP, N-terminal pro–B-type natriuretic peptide; VEGF, vascular endothelial growth factor

### Clinical and laboratory features

Baseline characteristics are summarized in Table 1. Patients with concurrent amyloid deposition were older at diagnosis (median 62 years (range, 49–73) vs. 53 years (range, 29–71), *p* = 0.022), while sex distribution was comparable (male: 85.7% vs. 70.9%, *p* = 0.408). Syndrome-defining manifestations were broadly similar between groups, with no statistically significant differences across individual POEMS features (Table 1). Serous effusions were frequent in both groups (85.7% vs. 78.2%, *p* = 0.645). Pericardial effusion was numerically more common in the concurrent amyloid deposition group (85.7% vs. 54.5%), although the difference did not reach statistical significance (*p* = 0.115).

The monoclonal immunoglobulin profile differed between groups. The proportion of IgA isotype tended to be higher in the concurrent amyloid deposition group (85.7% vs. 48.0%, *p* = 0.061), and IgA levels were significantly higher (median 6.21 g/L (range, 0.07–9.89) vs. 2.17 g/L (range, 0.38–19.10), *p* = 0.034). Lambda free light chains were markedly increased in the concurrent amyloid deposition group (median 127.0 mg/L (range, 57.8–4861.0) vs. 35.4 mg/L (range, 9.5–1376.8), *p* < 0.001), whereas IgG levels were similar (8.9 g/L (range, 5.9–23.9) vs. 10.3 g/L (range, 3.0–30.9), *p* = 0.335).

Routine hematologic and hepatic indices were not significantly different, including hemoglobin (108 g/L (range, 93–160) vs. 128 g/L (range, 76–170), *p* = 0.382) and ALT (14 U/L (range, 4–26) vs. 11 U/L (range, 4–43), *p* = 0.556). Platelet counts showed a numerical difference (195 × 10^9^/L (range, 99–422) vs. 276.5 × 10^9^/L (range, 21–592), *p* = 0.088). C-reactive protein (CRP) levels did not differ significantly between groups, with median values within the reference range (1.1 mg/L (range, 0.4–2.7) vs. 2.6 mg/L (range, 0.2–266.6), *p* = 0.340). Renal function parameters showed a trend toward greater impairment in the concurrent amyloid deposition group, with higher creatinine (104 µmol/L (range, 41–133) vs. 77 µmol/L (range, 31–1037), *p* = 0.067) and lower eGFR (61.1 mL/min/1.73 m² (range, 49.7–124.9) vs. 98.0 (range, 4.5–149.3), *p* = 0.054), although these differences did not reach statistical significance. Markers of cardiac stress and injury were substantially higher in the concurrent amyloid deposition group, with NT-proBNP having a median of 1250 pg/mL (range, 159–20510) compared to 359 pg/mL (range, 5–2512) (*p* = 0.009), and hs-cTnI having a median of 47.4 pg/mL (range, 1.9–3307.6) compared to 2.1 pg/mL (range, 1.9–21.7) (*p* = 0.002).

### Cardiac structure and function

Echocardiographic parameters are summarized in Table 2. Left atrial diameter (LA) and left ventricular end-diastolic dimension (LVEDD) were similar between groups (LA, median 35 mm (range, 31–40) vs. median 33 mm (range, 24–44), *p* = 0.246; LVEDD, median 46.5 mm (range, 42–55) vs. median 46.5 mm (range, 31–56), *p* = 0.929). In contrast, the concurrent amyloid deposition group had greater left ventricular wall thickness, with higher posterior wall thickness (LVPW, median 12 mm (range, 9–15) vs. 10 mm (range, 8–13), *p* = 0.006) and interventricular septal thickness (IVS, median 12.5 mm (range, 9–18) vs. 10 mm (range, 7–14), *p* = 0.011). Left ventricular ejection fraction did not differ significantly (63.5% (range, 48–78) vs. 66.5% (range, 55–75), *p* = 0.462).

**Table 2:** Echocardiographic and tissue Doppler imaging parameters according to concurrent amyloid deposition status

Parameter	Concurrent amyloid deposition (*n* = 7)	POEMS-only (*n* = 55)	*p* value
**Standard 2D echocardiography**			
LA, mm	35 (31–40)	33 (24–44)	0.246
LVEDD, mm	46.5 (42–55)	46.5 (31–56)	0.929
LVPW, mm	12 (9–15)	10 (8–13)	**0.006**
IVS, mm	12.5 (9–18)	10 (7–14)	**0.011**
LVEF, %	63.5 (48–78)	66.5 (55–75)	0.462
E, cm/s	75 (39–119)	72.5 (36–115)	0.772
A, cm/s	79.5 (57–114)	79 (49–147)	0.961
E/A ratio	0.78 (0.42–2.04)	0.85 (0.40–1.78)	0.430
**TDI (septal mitral annulus)**			
S′, cm/s	7.5 (2–9)	9 (4–14)	0.078
E′, cm/s	4.5 (3–7)	8 (3–16)	**0.008**
A′, cm/s	9 (3–9)	11 (5–14)	**0.006**

Values are presented as median (range). All measurements were obtained at baseline before systemic therapy

Abbreviations: A, late diastolic transmitral inflow peak velocity; A′, late diastolic mitral annular velocity by TDI; E, early diastolic transmitral inflow peak velocity; E′, early diastolic mitral annular velocity by TDI; IVS, interventricular septum; LA, left atrial diameter; LVEDD, left ventricular end-diastolic dimension; LVEF, left ventricular ejection fraction; LVPW, left ventricular posterior wall thickness; S′, systolic mitral annular velocity by TDI; TDI, tissue Doppler imaging

Transmitral inflow indices were not significantly different (E-wave: *p* = 0.772; A-wave: *p* = 0.961; and E/A: *p* = 0.430). However, tissue Doppler assessment at the septal mitral annulus showed lower velocities in the concurrent amyloid deposition group, including reduced early diastolic velocity (E′, 4.5 cm/s (range, 3–7) vs. 8 cm/s (range, 3–16), *p* = 0.008) and late diastolic velocity (A′, 9 cm/s (range, 3–9) vs. 11 cm/s (range, 5–14), *p* = 0.006). The systolic annular velocity (S′) also tended to be lower (7.5 cm/s (range, 2–9) vs. 9 cm/s (range, 4–14), *p* = 0.078). Overall, these findings were consistent with more pronounced cardiac involvement in the concurrent amyloid deposition group.

### Treatment regimens and responses

Forty-eight patients received standardized chemotherapy (concurrent amyloid deposition, *n* = 5; POEMS-only, *n* = 43), while 14 received supportive treatment only (concurrent amyloid deposition, *n* = 2; POEMS-only, *n* = 12) (Supplementary Table 1). Among patients receiving standardized chemotherapy, first-line regimens in the POEMS-only group included bortezomib, lenalidomide, and dexamethasone (VRD, *n* = 10), bortezomib and dexamethasone (VD, *n* = 14), lenalidomide and dexamethasone (RD, *n* = 18), and cyclophosphamide and dexamethasone (CTX + dexamethasone, *n* = 1), whereas those with concurrent amyloid deposition received bortezomib and dexamethasone (VD, *n* = 2), lenalidomide and dexamethasone (RD, *n* = 2), or CD38-targeted therapy combined with bortezomib and dexamethasone (CD38 + VD, *n* = 1).

**Fig. 2:**
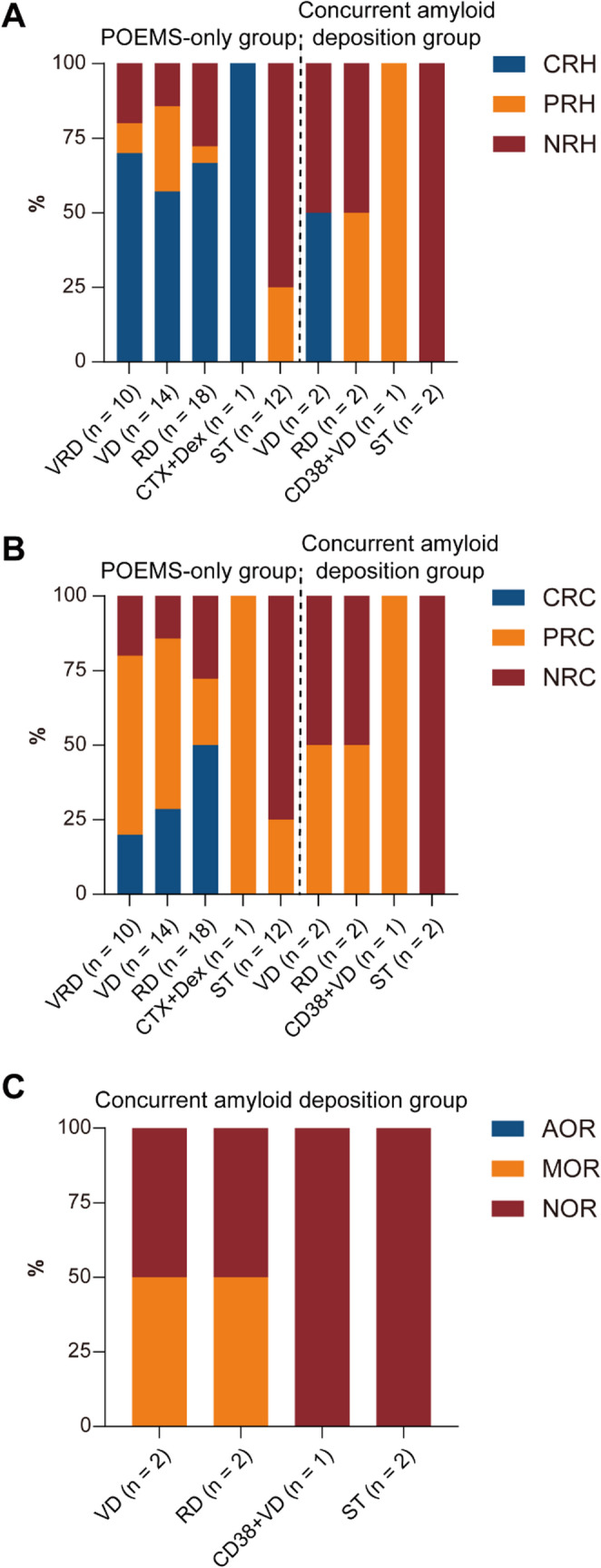
Response distributions by treatment regimen in patients with POEMS syndrome with or without concurrent amyloid deposition. (**A**, **B**) Hematologic and clinical response distributions in the POEMS-only group and the concurrent amyloid deposition group according to treatment regimens: (**A**) hematologic response, (**B**) clinical response. (**C**) Organ response distributions by treatment regimen in the concurrent amyloid deposition group. Abbreviations: AOR, all-organ response; CRC, complete clinical response; CRH, complete hematologic response; MOR, mixed organ response; NOR, no organ response; NRC, no clinical response; NRH, no hematologic response; PRC, partial clinical response; PRH, partial hematologic response. VRD, bortezomib/lenalidomide/dexamethasone; VD, bortezomib/dexamethasone; RD, lenalidomide/dexamethasone; CTX, cyclophosphamide; CD38 + VD, anti-CD38 monoclonal antibody plus VD; ST, supportive treatment

Response outcomes are summarized in Fig. 2 and Supplementary Table 1. Overall hematologic and clinical responses of standardized chemotherapy were numerically lower in the concurrent amyloid deposition group than in the POEMS-only group (3/5 vs. 34/43, Supplementary Table 1), given the small sample size and regimen heterogeneity in the concurrent amyloid deposition group, these comparisons should be regarded as descriptive. Within the POEMS-only group, patients treated with bortezomib-containing regimens achieved CRH and CRC rates of 62.5% (15/24) and 25.0% (6/24), respectively. By comparison, patients treated with lenalidomide without bortezomib achieved CRH and CRC rates of 66.7% (12/18) and 50.0% (9/18), respectively, which were numerically higher than those observed with bortezomib-containing regimens. Cardiac organ response (AOR/MOR/NOR) in the concurrent amyloid deposition group is shown in Fig. 2C, stratified by treatment approach when applicable. Overall, organ improvement in this subgroup was limited.

Two patients in the POEMS-only group achieved deep hematologic responses after chemotherapy followed by autologous stem cell transplantation. A third patient, treated with CAR-T therapy, attained CRH but later succumbed to a severe infection, underscoring the treatment-related competing risks in highly selected patients.

### Survival analysis and prognostic factors

**Fig. 3:**
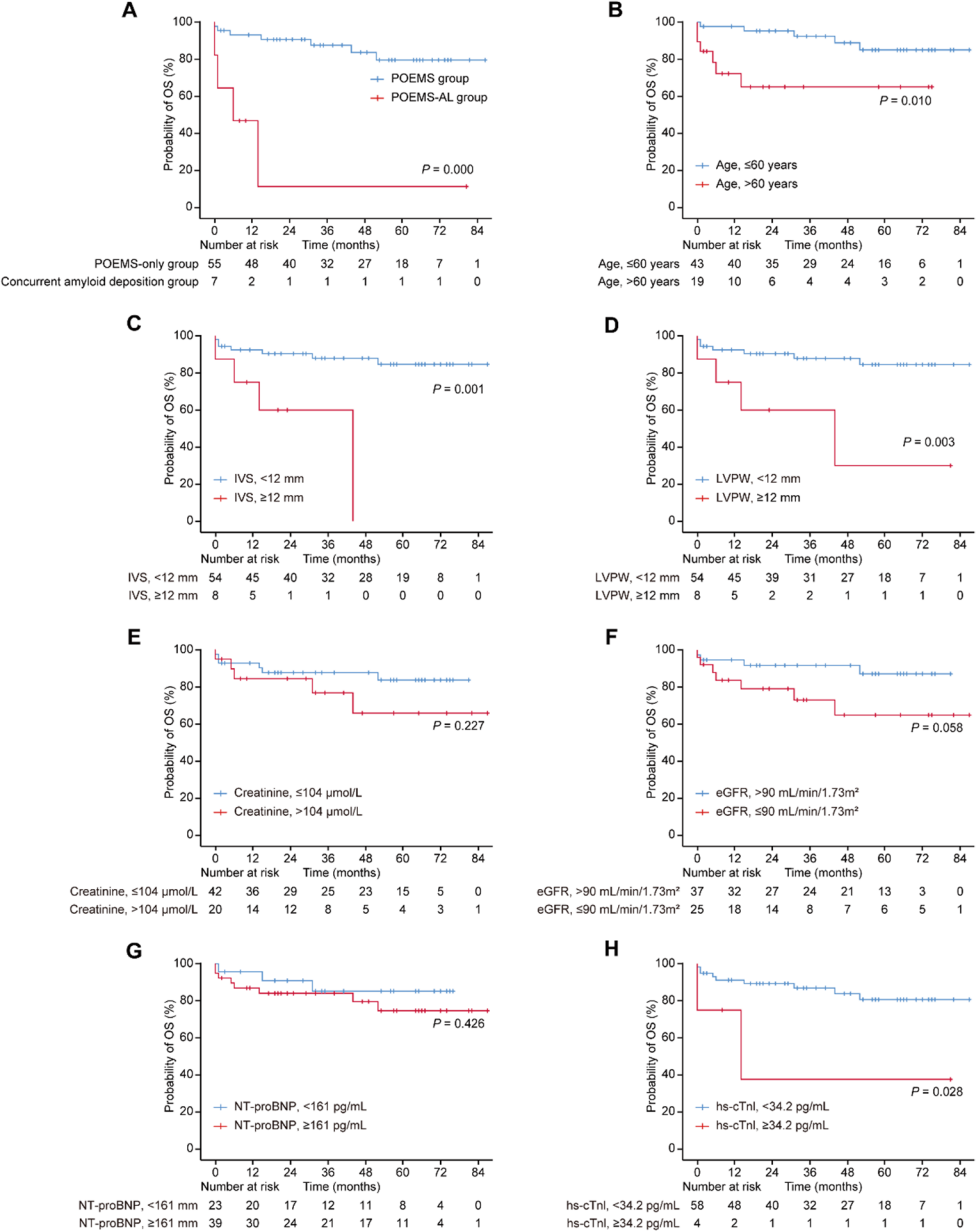
Kaplan–Meier overall survival (OS) curves stratified by diagnosis and baseline variables. Overall survival was stratified by (**A**) diagnosis (POEMS-only group vs. concurrent amyloid deposition group), (**B**) age (≤ 60 vs. > 60 years), (**C**) interventricular septal thickness (IVS; < 12 vs. ≥ 12 mm), (**D**) left ventricular posterior wall thickness (LVPW; < 12 vs. ≥ 12 mm), (**E**) creatinine (≤ 104 vs. > 104 µmol/L), (**F**) estimated glomerular filtration rate (eGFR; > 90 vs. ≤ 90 mL/min/1.73 m^2^), (**G**) NT-proBNP (< 161 vs. ≥ 161 pg/mL), and (**H**) high-sensitivity cardiac troponin I (hs-cTnI; < 34.2 vs. ≥ 34.2 pg/mL). P values were calculated by the log-rank test

After a median follow-up of 53.0 months (95% CI, 42.0–64.1), there were 11 deaths (concurrent amyloid deposition: 4/7; POEMS-only: 7/55). Median OS was not reached and the 3-year OS rate was 51.6%. Kaplan–Meier analysis showed inferior overall survival (OS) in the concurrent amyloid deposition group compared with POEMS-only (log-rank *p* < 0.001) (Fig. 3A). Older age (> 60 years) was associated with worse OS (*p* = 0.010) (Fig. 3B). Increased left ventricular wall thickness was also associated with differences in overall survival, as IVS ≥ 12 mm (*p* = 0.001) and LVPW ≥ 12 mm (*p* = 0.003) were each associated with inferior survival (Fig. 3C–D).

Renal indices were not significantly associated with OS by Kaplan-Meier analysis (Fig. 3E-F). NT-proBNP dichotomized at 161 pg/mL and hs-cTnI dichotomized at 34.2 pg/mL (laboratory upper reference limits) were evaluated; NT-proBNP was not associated with OS (*p* = 0.426), whereas hs-cTnI was associated with inferior survival (*p* = 0.028) (Fig. 3G-H).

**Fig. 4:**
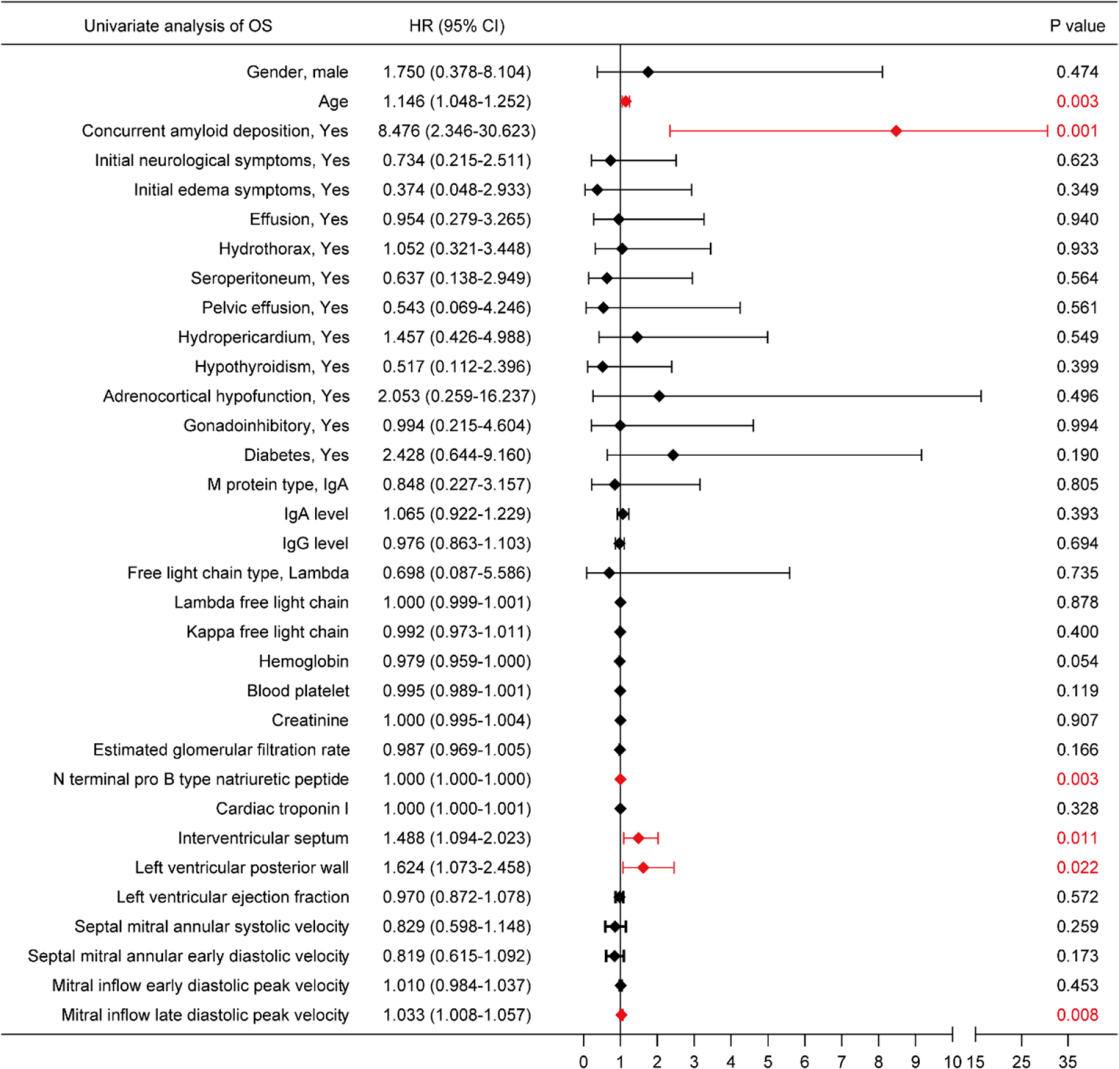
Forest plot of univariate Cox regression analyses for overall survival (OS). Hazard ratios and 95% confidence intervals from univariate Cox proportional hazards regression are shown. Abbreviations: CI, confidence interval; HR, Hazard ratio; IgA, immunoglobulin A; OS, overall survival

In univariate Cox regression (Fig. 4), age (HR 1.146, 95% CI 1.048–1.252; *p* = 0.003) and concurrent amyloid deposition (HR 8.476, 95% CI 2.346–30.623; *p* = 0.001) were associated with increased mortality risk. Cardiac structural and functional measures were also associated with OS, including IVS thickness (HR 1.488, 95% CI 1.094–2.023; *p* = 0.011), LVPW thickness (HR 1.624, 95% CI 1.073–2.458; *p* = 0.022), and mitral inflow late diastolic peak velocity (HR 1.033, 95% CI 1.008–1.057; *p* = 0.008). Given the small number of events, multivariable modeling was not performed to avoid overfitting.

## Discussion

In this single-center cohort, biopsy-proven concurrent amyloid deposition was identified in a subset of patients with POEMS syndrome and was associated with a distinct clinical profile and worse outcomes. Compared with POEMS-only, patients with concurrent amyloid deposition were older, had higher IgA levels and a greater circulating light-chain burden, and showed more pronounced cardiac involvement reflected by higher biomarkers, greater wall thickness, and lower tissue Doppler velocities. Responses appeared numerically lower in this subgroup, and overall survival was inferior compared with POEMS-only.

POEMS syndrome and AL amyloidosis both arise from clonal plasma cell disorders but differ in downstream pathophysiology. In POEMS, cytokine dysregulation—particularly VEGF—drives endothelial activation, vascular permeability, and volume overload [18]. In AL amyloidosis, organ dysfunction results primarily from light-chain amyloid deposition and direct proteotoxicity [19]. When these processes coexist, VEGF-driven volume overload may mask or amplify amyloid-related organ injury, potentially contributing to delayed recognition and adverse outcomes.

Cardiac involvement emerged as the central clinical feature distinguishing the concurrent amyloid deposition group from POEMS-only in our cohort. These patients had higher cardiac biomarkers, thicker ventricular walls, and lower tissue Doppler velocities, consistent with more severe myocardial involvement. Because VEGF-mediated extravascular volume overload in POEMS can contribute to apparent wall thickening and biomarker elevation, a multimodal evaluation integrating biomarkers, echocardiography (including tissue Doppler), and, when available, cardiac magnetic resonance imaging is important for contextualizing cardiac abnormalities and improving diagnostic confidence [10, 20].

The immunophenotype of this subgroup also warrants attention. Similar to classic POEMS, the paraprotein was typically lambda-restricted, but the magnitude of circulating light chains and biomarker perturbation was greater, suggesting a clinically relevant increase in amyloid burden and/or a more amyloidogenic clone in this subset.

Therapeutic responses appeared numerically lower in patients with concurrent amyloid deposition, consistent with the prognostic weight of cardiac involvement in systemic amyloidosis and related overlap states [21]. Although treatment implications should be drawn cautiously, these findings suggest that patients with POEMS syndrome and coexisting amyloid deposition may require prompt plasma cell-directed therapy together with careful cardiac and volume management. In selected patients, intensified plasma cell-directed approaches (including anti-CD38 antibodies) may be considered, recognizing competing risks and the need for careful patient selection [22, 23].

Compared with patients with concurrent amyloid deposition, those in the POEMS-only group showed numerically better responses to chemotherapy. Within the POEMS-only cohort, bortezomib-sparing regimens were associated with numerically higher hematologic and clinical complete response rates than bortezomib-containing regimens; however, given the retrospective, non-randomized design and limited sample size, this finding should be interpreted cautiously. As neuropathy is a defining feature of POEMS and bortezomib may aggravate peripheral neuropathy, treatment selection and tolerability could have influenced both treatment exposure and response [24]. Bortezomib-sparing approaches may be clinically appealing in selected patients, but prospective data are needed to define the optimal regimen. In addition, small retrospective series have reported clinical activity of anti-CD38 therapy in refractory POEMS [25, 26]. Future studies could further evaluate anti-CD38–based combinations, including with lenalidomide, in POEMS patients with and without concurrent amyloid deposition.

From a diagnostic standpoint, distinguishing VEGF-mediated volume overload from amyloid cardiomyopathy is challenging but clinically essential. Cardiac MRI and radionuclide imaging can provide incremental value, yet tissue confirmation remains important when clinical suspicion is high [27]. In practice, disproportionate elevations of NT-proBNP or troponin, progressive wall thickening despite improvement in volume status, low-voltage ECG, or new arrhythmias should prompt evaluation for amyloidosis in patients with POEMS [28].

Clinical implications for evaluation and monitoring. In routine practice, concurrent amyloid deposition should be suspected in older patients with POEMS syndrome who have a higher lambda free light-chain burden and/or an IgA paraprotein, particularly when NT-proBNP or hs-cTnI is disproportionately elevated. A systematic baseline cardiac assessment—ECG, transthoracic echocardiography with tissue Doppler, and serial biomarker monitoring—can help define risk and identify patients who warrant escalation to cardiac MRI and/or nuclear imaging where available. In the presence of red-flag cardiac findings or otherwise unexplained organ dysfunction, tissue confirmation of amyloid deposition remains important, with amyloid typing pursued whenever feasible [29]. When concurrent amyloid deposition is confirmed or strongly suspected, management should prioritize rapid plasma cell cytoreduction alongside early cardio-hematology co-management to optimize heart failure therapy, rhythm surveillance, and volume status.

These findings also inform longitudinal monitoring. In POEMS, hematologic improvement may precede resolution of edema and other organ manifestations, whereas in patients with concurrent amyloid deposition, serial cardiac biomarkers and echocardiographic parameters can provide early signals of ongoing cardiac risk even when hematologic responses are achieved. In our survival analyses, hs-cTnI showed a stronger association with overall survival than NT-proBNP. Because NT-proBNP is largely cleared by the kidneys and is inversely correlated with eGFR [30], elevated NT-proBNP may reflect not only cardiac dysfunction but also impaired renal function, potentially attenuating its association with overall survival. These findings suggest that hs-cTnI may be a more informative biomarker for risk assessment in this cohort, and closer cardiology follow-up with attention to hs-cTnI may be helpful.

Several limitations should be acknowledged. The retrospective, single-center design introduces selection bias and limits generalizability. Importantly, amyloidosis was biopsy-confirmed rather than systematically screened in all patients, and therefore the observed proportion of concurrent amyloid deposition in this cohort may not reflect true prevalence. The concurrent amyloid deposition subgroup was small, precluding multivariable adjustment and limiting power for subgroup analyses. In addition, comprehensive amyloid fibril typing (e.g., by mass spectrometry) was not routinely available; thus, the amyloid subtype could not be definitively assigned. Although all cases were biopsy-proven and arose in the setting of a monoclonal plasma cell disorder, misclassification cannot be excluded. POEMS is characterized by overexpression of proinflammatory cytokines; accordingly, in the 7 patients with concurrent amyloid deposition—particularly the 3 with renal involvement—AA amyloidosis remained an important differential diagnosis [1, 31]. Because serum amyloid A testing was not available, this possibility could not be formally excluded [32]. The absence of marked CRP elevation in these patients may be viewed as supportive context against active inflammatory AA amyloidosis, but it is not sufficient to establish amyloid subtype [33]. These limitations should be considered when interpreting the present findings.

Future studies should prioritize multi-institutional collaboration and prospective registries to better define incidence, optimal diagnostic algorithms, and treatment strategies for POEMS syndrome with concurrent amyloid deposition. Incorporation of standardized cardiac imaging and biomarker trajectories, as well as evaluation of novel plasma cell–directed therapies and fibril-directed approaches, may further improve risk stratification and outcomes [34, 35].

In conclusion, POEMS syndrome with biopsy-proven concurrent amyloid deposition represents an overlap phenotype associated with disproportionate cardiac involvement and worse survival. Heightened clinical suspicion, early recognition, and coordinated, organ-focused multidisciplinary management are essential to improve outcomes.

## Supplementary Information

Below is the link to the electronic supplementary material.

Supplementary File 1 (DOCX 304 KB)

## Data Availability

To protect participant privacy, individual-level data are not publicly available. De-identified data that support the findings of this study (including the data underlying the figures and tables) are available from the corresponding author upon reasonable request and subject to institutional approval.
