# Supplementary material for: Clinical impact and prognostic implications of concurrent amyloid deposition in patients with POEMS syndrome: A single-center retrospective study
Source: Ann Hematol. 2026 Apr 9;105(5):235. doi: 10.1007/s00277-026-06995-1 (PMC13065611; doi:10.1007/s00277-026-06995-1)
Supplement: Supplementary file 1 — (DOCX 304 KB) [file 277_2026_6995_MOESM1_ESM.docx]

**Supplementary Figure 1. Flow diagram of patient selection.**


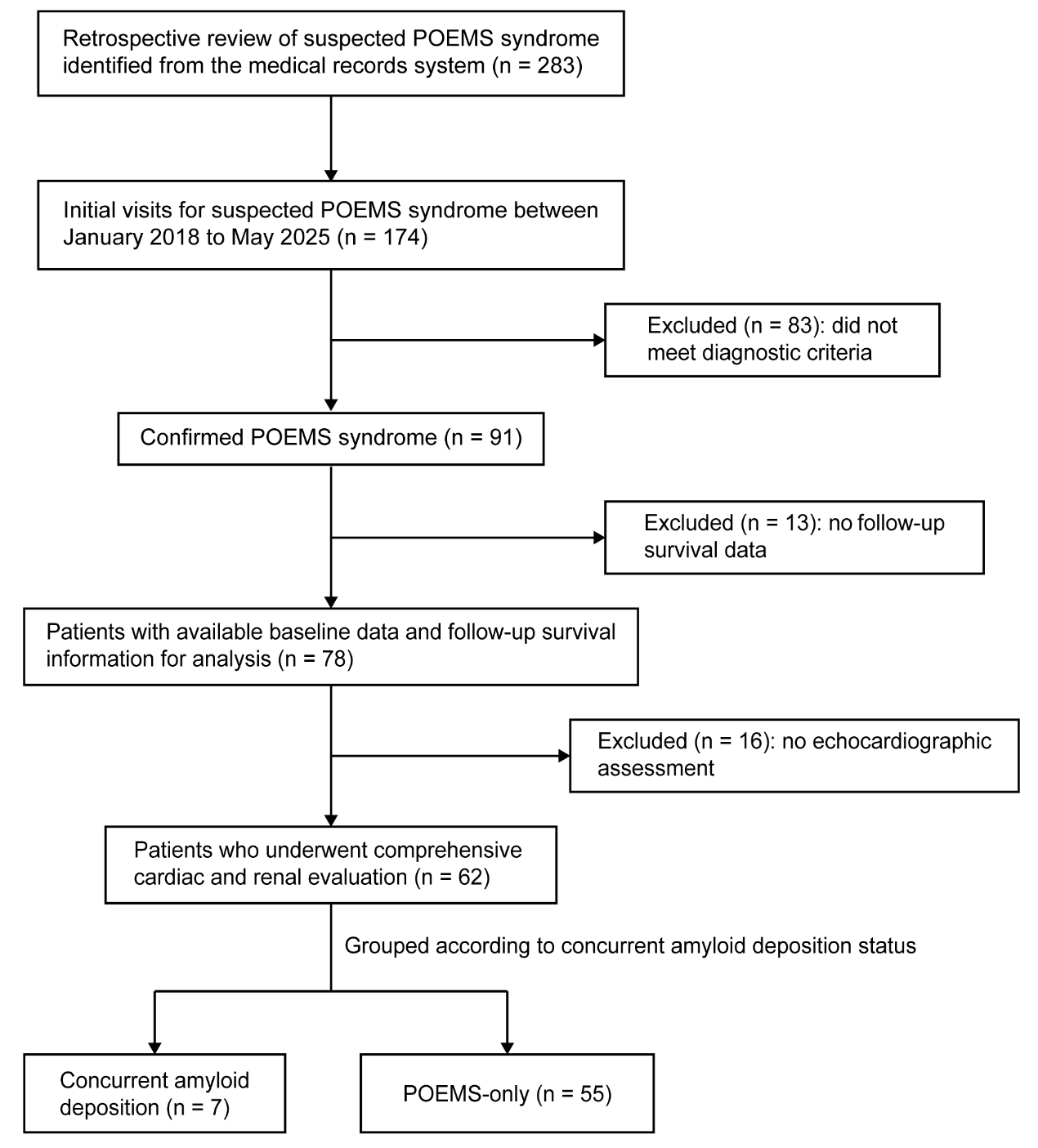


Flow diagram illustrating patient screening and final cohort selection.

Abbreviations: n, number.

**Supplementary Table 1.** **Best responses by treatment category.**

|  | **Concurrent amyloid deposition (n=7)** | | **POEMS-only (n=55)** | |
| --- | --- | --- | --- | --- |
|  | standardized chemotherapy  (n=5) | supportive treatment only (n=2) | standardized chemotherapy  (n=43) | supportive treatment only (n=12) |
| **Best hematological response** |  |  |  |  |
| CRH | 1/5 | 0/2 | 28/43 | 0/12 |
| PRH | 2/5 | 0/2 | 6/43 | 3/12 |
| NRH | 2/5 | 2/2 | 9/43 | 9/12 |
| Overall hematologic response | 3/5 | 0/2 | 34/43 | 3/12 |
| **Best clinical response** |  |  |  |  |
| CRC | 0/5 | 0/2 | 15/43 | 0/12 |
| PRC | 3/5 | 0/2 | 19/43 | 3/12 |
| NRC | 2/5 | 2/2 | 9/43 | 9/12 |
| Overall clinical response | 3/5 | 0/2 | 34/43 | 3/12 |
| **Best organ response** |  |  |  |  |
| AOR | 0/5 | 0/2 | - | - |
| MOR | 2/5 | 0/2 | - | - |
| NOR | 3/5 | 2/2 | - | - |

‘-’ indicates not applicable.

Abbreviations: AOR, all-organ response; CRC, complete clinical response; CRH, complete hematologic response; MOR, mixed organ response; NOR, no organ response; NRC, no clinical response; NRH, no hematologic response; PRC, partial clinical response; PRH, partial hematologic response.

**Supplementary Table 2. Abbreviations table.**

| A′ | Late diastolic mitral annular velocity by tissue Doppler imaging. |
| --- | --- |
| AL | Immunoglobulin light-chain (AL) amyloidosis. |
| AOR | All-organ response. |
| A-wave | Mitral inflow late diastolic peak velocity. |
| CRC | Complete clinical response. |
| CRH | Complete hematologic response. |
| E′ | Early diastolic mitral annular velocity by tissue Doppler imaging. |
| ECG | Electrocardiogram. |
| eGFR | Estimated glomerular filtration rate. |
| E-wave | Mitral inflow early diastolic peak velocity. |
| HR | Hazard ratio. |
| hs-cTnI | High-sensitivity cardiac troponin I. |
| IVS | Interventricular septum. |
| LA | Left atrial diameter. |
| LVEDD | Left ventricular end-diastolic dimension. |
| LVEF | Left ventricular ejection fraction. |
| LVPW | Left ventricular posterior wall thickness. |
| MOR | Mixed organ response. |
| NOR | No organ response. |
| NRC | No clinical response. |
| NRH | No hematologic response. |
| NT-proBNP | N-terminal pro–B-type natriuretic peptide. |
| OS | Overall survival. |
| PRC | Partial clinical response. |
| PRH | Partial hematologic response. |
| S′ | Systolic mitral annular velocity by tissue Doppler imaging. |
| VEGF | Vascular endothelial growth factor. |
